# Artificial intelligence in cardiology: implications for healthcare outcomes

**DOI:** 10.3389/frai.2026.1818586

**Published:** 2026-07-15

**Authors:** Mikayla N. Harris, Parth Desai, Eric Yalley, Yuxuan Wu, Michael Singleton, Ella DeBerry, Yashvardhan Batta, Gal Levy, Georges E. Haddad

**Affiliations:** 1 Department of Physiology and Biophysics, College of Medicine, Howard University, Washington, DC, United States; 2 Department of Internal Medicine, Temple University, Philadelphia, PA, United States; 3 Department of Surgery, College of Medicine, Howard University, Washington, DC, United States

**Keywords:** artificial intelligence, bias, cardiology, diagnosis, health care outcome

## Abstract

Artificial Intelligence (AI) has the potential to revolutionize medicine, particularly in the field of cardiology. There are significant diagnostics and treatments variabilities in the field of cardiovascular medicine that affects racial and ethnic racially and ethnically diverse populations as well as female patients across all age groups. The efforts put forth towards the development of AI and precision medicine within the cardiovascular practice do not fully account for existing variations in cardiovascular care delivery. AI models and precision medicine tools that were created with uncomprehensive data primarily drawn from White populations risk embedding historical differences into clinical decision-support systems. This paper outlines the integrative approach taken to review the current variabilities that persist within younger adults (<65 years) and older adults (≥ 65 years) who have cardiovascular disease. Additionally, genetic factors, limited access to care, health literacy, lack of insurance coverage and adherence are examined, as these are frequently cited as major contributors to health care adverse outcomes but remain under-researched and unresolved even with the expansion of Medicaid. For instance, Black patients experience higher prevalence of heart failure (HF) and hypertension, especially transthyretin amyloid cardiomyopathy HF, with Black women being disproportionately affected due to higher structural, environmental and clinical factors. Also, racially and ethnically diverse children with CVDs have higher odds of mortality than their White counterparts. The integration of AI in cardiovascular medicine must first be preceded by an active effort to restructure systems and reduce variable outcomes. Future research must prioritize diverse genomic datasets and equitable comprehensive representation in clinical trials. These initiatives are better served if they are driven by institutions that historically serve racially and ethnically diverse populations and communities to better enhance inclusion and fairness in electronic medical record keeping. Accordingly, cardiovascular medical practices and technology can progress forward with AI and precision medicine models that are both equitable and accurate.

## Introduction

Cardiovascular disease remains the leading cause of death in the United States, and although overall mortality has declined since the 2000s, the burden is not evenly distributed (Heron and Anderson, 2016). Black and Hispanic patients experience a disproportionate burden of cardiovascular disease, with underutilization of evidence-based therapies and higher procedural and mortality risks. Black patients have the highest lifetime risk of peripheral arterial disease (30% vs. 19% in White and 22% in Hispanic patients) and the highest overall prevalence of heart failure (Virani et al., 2021). Healthcare disparities refer to systematic inequities in access to care, quality of treatment, and clinical outcomes across patient groups that frequently align with racial, ethnic, socioeconomic, and gender boundaries. Within cardiology, these inequities manifest as higher burdens of cardiovascular disease, delayed diagnosis, limited access to subspecialty services, and disproportionately worse outcomes for underserved populations. Factors driving these disparities include limited healthcare access, lower health literacy, genetic risk factors, and inequities in treatment delivery, including gender and race bias. Addressing these disparities is essential before evaluating how artificial intelligence (AI) can be implemented equitably in cardiovascular care. This requires an understanding of AI and its growing role in modern healthcare.

**Figure 1:**
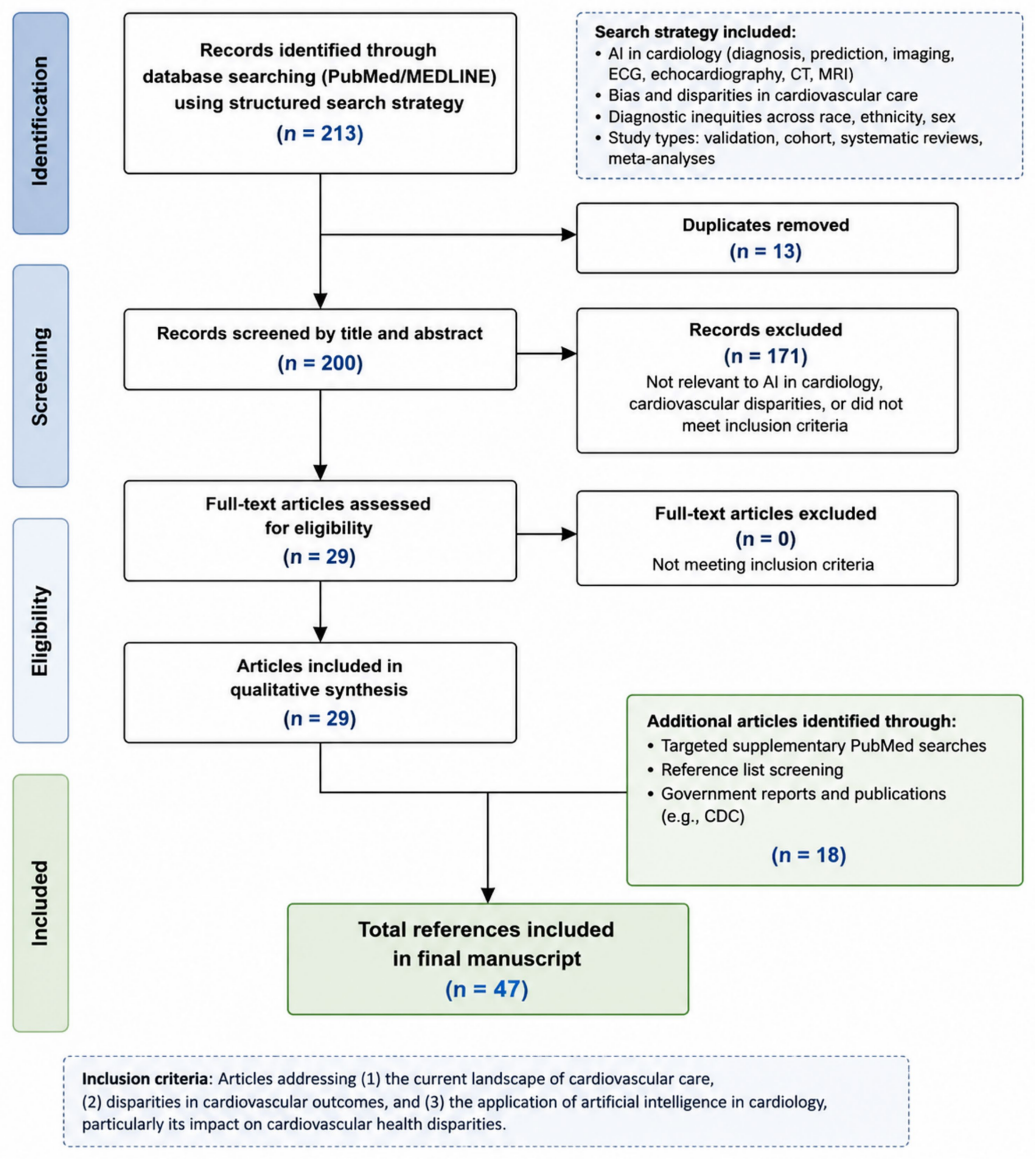
PRISMA-inspired flow diagram of literature search and study selection for artificial intelligence in cardiology and cardiovascular health disparities.

Artificial intelligence is a broad domain of computer science encompassing diverse methods that integrate human-like intelligence with large-scale data. Its aims include streamlining routine clinical workflows and enhancing the accuracy of data-driven decision-making. With ongoing improvements in the availability and quality of disease data, interest in implementing AI modes across healthcare has grown. However, in the context of persistent racial and socioeconomic disparities in cardiovascular care, the rapid adoption of AI in cardiology raises critical questions about whether these technologies will mitigate or instead exacerbate existing inequities. This review examines how such disparities may influence the development, performance, and equity of AI-assisted healthcare Figure 1.

## Methods

In this narrative review, we conducted an analysis of relevant articles pertaining to cardiovascular disease disparities and the current use of AI in cardiology. Literature was searched on PubMed and government reports and publications including the Center for Disease Control and Prevention (CDC). Core AI cardiovascular literature was identified using (“artificial intelligence” OR “machine learning” OR “deep learning”) AND (cardiology OR cardiovascular) AND (diagnosis OR prediction OR imaging OR ECG OR echocardiography OR CT OR MRI), with refinement for evidence strength using (validation OR cohort OR systematic review OR meta-analysis). Studies addressing bias and disparities used (“artificial intelligence” OR “machine learning”) AND (bias OR fairness OR disparity OR inequity OR racism OR “health disparities”) AND (cardiology OR cardiovascular). Diagnostic inequities were captured using (cardiovascular OR “heart disease”) AND (diagnostic error OR misdiagnosis OR delayed diagnosis OR underdiagnosis) AND (race OR ethnicity OR sex OR disparities). Overall, 213 records were identified through database searching. Following removal of 13 duplicates and screening of titles and abstracts, 29 full-text articles met inclusion criteria and were included in the qualitative synthesis. Additional relevant articles identified through targeted supplementary PubMed searches and reference list screening were also incorporated to ensure comprehensive coverage of key concepts. Overall, 47 references were included in the final manuscript. Full text articles were included if they addressed the current landscape of cardiovascular care, disparities in cardiovascular outcomes, as well as the application of artificial intelligence in cardiology, particularly its impact on cardiovascular health disparities.

## AI applications in cardiovascular diagnostics

The field of cardiology has made substantial progress in diagnostic capabilities and continues to advance as new research expands the field. Challenges in diagnosis, treatment, and prevention still exist. Pope et al. revealed that among patients with acute cardiac ischemia or unstable angina, approximately 2% of the patients were mistakenly discharged from the emergency department. This number is seemingly low, however the likelihood of a missed diagnosis is significantly higher in women under 55 (odds ratio (OR) for discharge, 6.7; 95%CI, 1.4–32.5), nonwhite patients (OR, 2.2; 95% CI, 1.1–4.3), those with presenting symptoms of shortness of breath (OR, 2.7; 1.1 to 6.5) or those with a normal or nondiagnostic ECG (OR, 3.3; 95% CI, 1.7 to 6.3) (Hector Pope et al., 2000). This highlights that classic diagnostic tools such as ECGs and symptomology require further workup in female and non-White patients to provide accurate diagnosis of acute coronary syndrome. Without understanding this, patients may be misdiagnosed. This data can be further compounded by persistent diagnostic disparities (Damrauer et al., 2019; Coniglio et al., 2022). Similar patterns of delayed or missed diagnosis are observed in other cardiovascular conditions such as cardiac amyloidosis. This pathology is characterized by nonspecific or heterogeneous presentation which frequently overlaps with conditions such as hypertensive heart disease (Rapezzi et al., 2015). As a result, there are diagnostic delays which postpone referral, genetic testing, and initiation of disease-modifying therapies, often resulting in more advanced heart failure at presentation and poorer prognosis. In response to these persistent diagnostic gaps, attention has increasingly turned to artificial intelligence–driven machine learning approaches—particularly those leveraging electrocardiography and cardiac imaging, to enhance early detection and risk stratification. However, to meaningfully reduce disparities, such tools must be further developed and validated to represent underserved populations to avoid perpetuating existing inequities in cardiovascular care.

## Machine learning and predictive analytics

Machine learning and predictive analytics may streamline cardiovascular care by enhancing diagnostic accuracy, improving risk stratification, and facilitating earlier identification of cardiovascular disease. Prior systematic reviews and meta-analyses suggest that machine learning algorithms may provide clinically useful predictive performance for outcomes such as coronary artery disease, myocardial infarction, heart failure and arrhythmia, although methodological heterogeneity and concerns regarding generalizability remain important limitations (Friedrich et al., 2021; Quer et al., 2021; Krittanawong et al., 2020).

In a recent study, two random forest machine learning models were evaluated to predict the risk of acute myocardial infarction (AMI) and ischemic heart disease (IHD) in cardiovascular patients. The AMI model demonstrated an accuracy of 0.97, sensitivity of 0.67, specificity of 1.00, and precision of 0.99. Similarly, the IHD-model showed comparable predictive performance with anticoagulant or antiplatelet use, and elevations in systolic blood pressure, mean blood glucose, and eGFR identified as major contributing variables (Salet et al., 2024).

Similarly to several emerging risk assessment models, the random forest algorithms did not incorporate race or social determinants of health into cardiovascular risk prediction, despite their well-established associations with cardiovascular disease. This may have influenced the performance of the models evaluated in the study, in the context of disproportionate burdens of hypertension, diabetes, and chronic kidney disease among patients of color.

Furthermore, a prospective study demonstrated potential utility of machine learning to enhance long-term risk prediction of myocardial infarction (MI) and cardiac death in asymptomatic patients. The machine learning model was trained on clinical variables, lipid profiles and cardiovascular risk factors, and imaging measures. The model demonstrated superior predictive performance compared with conventional atherosclerotic cardiovascular disease (ASCVD) risk assessment and coronary artery calcium scoring alone, supporting its potential value for improved cardiovascular risk stratification (Commandeur et al., 2020).

Although machine learning models are promising, the risk factors used by these models to predict outcomes may underrepresent a patient’s true risk. The NHANES III study evaluated metabolic abnormalities among minority populations compared to white participants using Glomerular filtration rate (GFR) as a reference. After adjustment for age, demographic factors, and GFR, Black and Hispanic participants were more likely than White participants to have elevated systolic blood pressure and abnormal electrolyte measures associated with declining kidney function, including phosphorus levels (Foley et al., 2007). This highlights the fact that standard chronic kidney disease thresholds may underestimate metabolic risk in minority patients; indicating that AI models using just GFR without including race and ethnicity to predict cardiovascular disease risk might be inaccurate. It is important to further initiatives to increase representation of minority ethnicities in data used to create AI and machine learning models. Ensuring representative data will reduce current disparities in CVD assessment and diagnosis in minority patients.

There are several publications which remain limited by retrospective study design, inadequate external validation, bias, highly selected patient cohorts, and real-world clinical utility. Some AI tools have emerged that demonstrate strong performance in research settings. As these AI tools undergo prospective multicenter validation and begin to be implemented into clinical settings, the functionality within diverse and underserved patient populations should be required as an assessment of their clinical effectiveness. While machine learning tools demonstrate substantial promise in predictive analytics, careful critical assessment of their clinical role, study methodology, quality and limitations as they relate to diverse populations remain essential.

The following sections examine the application of AI tools across commonly utilized cardiovascular diagnostic tools including electrocardiography, computerized, tomography, cardiac magnetic resonance imaging. For each diagnostic tool, we review the clinical application, machine, learning, tools utilized, as well as the impact on diagnostic accuracy, and clinical workflow. In evaluating the strengths and limitations of the study methods, design, and external validity we aim to emphasize the promise of these growing tools or concurrently highlighting the need for equitable, perspective generalizability prior to widespread clinical use.

## Electrocardiography (ECG)

Artificial intelligence-driven tools could serve as a promising modality for earlier and more accurate detection of cardiovascular disease. Al-Zaiti et al. conducted the first observational cohort study of its kind using a Random Forest (RF) AI model that reads ECG for diagnosis of Occlusion Myocardial Infarction (OMI) without ST elevation in patients presenting with chest pain (Al-Zaiti et al., 2023). The model was trained on 7,313 patients and validated on 3,827 patients. In this study, OMI was defined as a culprit coronary artery with TIMI flow grade 0–1 or TIMI grade 2 flow accompanied by severe coronary stenosis (>70%) and elevated peak fourth-generation troponin levels of 5–10 ng/mL. The RF model outperformed physicians and commonly used interpretation systems, such as the HEART score, in differentiating OMI from non-OMI patients (AUROC 0.87, 95% CI 0.85–0.90) and demonstrated a high ability to rule out OMI (NPV 0.993) (Al-Zaiti et al., 2023). However, this finding should be interpreted in the context of disease prevalence, as predictive values vary across clinical populations and additional performance beyond AUROC are necessary to assess clinical utility. Given that approximately 24%–35% of patients with NSTEMI per the study, may have underlying coronary occlusion requiring urgent catheterization, improved early detection of OMI could have important implications for reducing diagnostic delays and improving outcomes (Al-Zaiti et al., 2023). Additionally, this model was derived from patients with chest pain; however, the study does not address other presentations of OMI in underrepresented patient population.

Similarly, AI-enabled ECG models have been explored for the detection and prediction of atrial fibrillation (AF). Nasser et al. reviewed studies demonstrating that machine learning applied to standard ECGs and wearable devices can achieve high sensitivity and specificity for AF detection while potentially enabling earlier diagnosis and risk stratification. These technologies may expand continuous cardiovascular monitoring beyond traditional clinical settings, particularly through wearable and smartphone-based applications. However, the review emphasized challenges due to data bias, ECG quality variability, false positive and false negative classifications and reduced performance across demographic groups (Nasser et al., 2025). As with the OMI model, this study remains dependent on retrospective analyses, internally validated datasets or single-center cohorts, limiting conclusions regarding generalizability and implementation readiness. Prospective multicenter studies with external validation across diverse racial, ethnic and sex populations will be necessary to establish the validity, equity and real-world scalability of these AI-based cardiovascular diagnostic tools.

## Echocardiography

A deep-learning model trained on more than 14,000 echocardiograms automated multiple components of echocardiographic interpretation including view identification, chamber segmentation and disease detection. The model demonstrated high view-classification accuracy, which was 96% for parasternal long-axis images and produced automated measurements of cardiac structure, ejection fraction and strain that agreed with routine clinical and software measurements. Additional models were developed to detect hypertrophic cardiomyopathy, cardiac amyloid and pulmonary arterial hypertension with C statistics of 0.93, 0.87 and 0.85, respectively (Zhang et al., 2018). Because the model remained reliable across differences in image quality, patient factors, and machine vendors, it has strong potential for deployment in diverse, under-resourced settings. However, while the study demonstrated strong performance, external validation and multicenter participation is necessary to evaluate scalability for real-world use across diverse clinical settings. Among all imaging modalities, echocardiography is particularly vulnerable to operator-dependent acquisition variability, which has historically contributed to inconsistent measurements and diagnostic uncertainty in structurally marginalized populations. By automating view classification and quantitative measurements clinically, AI-assisted echocardiography may improve diagnostic workflows, reduce measurement variability, and enable earlier or more consistent detection of cardiac dysfunction. As a result, it may broaden access to high-quality imaging interpretation to busy clinics, emergency settings, and hospitals with limited cardiology expertise.

## Computerized tomography (CT)

An AI system trained on 1,636 cardiac CT scans and validated across 20,084 individuals drawn from four major cohorts (FHS, NLST, PROMISE, ROMICAT-II), produced calcium scores that were highly correlated with expert measurements (Spearman 0.92) and showed near-perfect test–retest reliability. The automated CAC scores consistently predicted cardiovascular events across diverse populations—heavy smokers, asymptomatic adults, stable chest pain patients, and acute chest pain presentations—with hazard ratios rising stepwise across CAC categories and performing equivalently to manual scoring. Because the model worked robustly across multiple institutions, scanner types, and both ECG-gated and non-gated CT, it demonstrated strong real-world generalizability (Zeleznik et al., 2021). Clinically, this approach could identify previously unrecognized cardiovascular risk from the millions of routine chest CTs performed annually, accelerate population-level risk stratification, and broaden access to proven imaging biomarkers—supporting earlier intervention and improving equity in cardiology prevention workflows. Although there is strong, multi-cohort validation, limitations remain important when interpreting these findings. While automated coronary artery, calcium scoring improves efficiency and risk prediction, further studies are required to determine how this detection tool improves long-term cardiovascular outcomes. Additionally, increased identification of incidental coronary calcification on routine chest CT may lead to overdiagnosis or reclassification of cardiovascular risk in some patients, potentially prompting additional diagnostic testing, specialist referrals, and healthcare utilization. The downstream clinical and economic consequences of these findings remain incompletely understood. Future studies should evaluate how automated CAC detection influences clinical decision-making compared with standard interpretation and whether it results in meaningful improvements in prevention strategies and patient outcomes, particularly among underserved populations who experience a disproportionate burden of cardiovascular disease.

## MRI

Automation of cardiac MRI interpretation has also become increasingly feasible through deep learning, targeting the long-standing barriers of expert dependence and time-intensive manual review in CMR workflows. Using data from 9,719 individuals across eight centers, the system screened for cardiac abnormalities and diagnosed 11 cardiovascular diseases with AUCs of 0.988 and 0.991, respectively, performing on par with experienced cardiologists and even surpassing them in diagnosing pulmonary arterial hypertension (Wang et al., 2024). Because the model was validated across multiple institutions, MRI vendors, and underserved populations, it showed strong generalizability beyond a single center. Clinically, this approach could accelerate CMR interpretation, reduce inter-reader variability, and broaden access to advanced imaging diagnostics in regions with limited CMR expertise. If prospectively validated across diverse, ethnic groups, these systems may expand, timely, screening and diagnosis. Additionally, screening in underserved populations improves the quality of advanced cardiac MRI imaging interpretation.

While AI-assisted MRI interpretation may improve diagnostic sensitivity, increased detection capabilities may increase the risk of incidental findings, over diagnosis, and increased testing burden. MRI studies frequently identify clinically uncertain abnormalities that do not represent serious diseases (O'Sullivan et al., 2018). The considerations underscore the importance of prospective evaluation of clinical utility, cost-effectiveness, and real word consequences of AI-MRI implementation.

## AI and healthcare access

Many rural and underserved U.S. counties lack cardiologists, with 1,454 of 3,143 counties (46.3%) having none, including 86.2% of rural counties. About 22 million people live in these areas, which generally have lower incomes, higher rates of uninsurance, limited primary care, and fewer resources like healthy food. Residents must travel much farther for care (87.1 vs. 16.3 miles), face higher cardiovascular risk (2.8 vs. 2.1), greater age-adjusted mortality (281.6 vs. 269.1 per 100,000), and shorter life expectancy, with Native Americans being the group most likely to live in these cardiologist-poor regions (Kim et al., 2024). Artificial intelligence has shown promising potential as a tool to reach underserved populations and extend healthcare access, particularly through telemedicine. Trivedi et al. (2025) conducted a study on the implementation of Coordinating Health Care with Artificial Intelligence-Supported Technology for Patients with Atrial Fibrillation (CHAT-AF). The conversational AI phone call system, CHAT-AF, recognizes patient speech, semantics, and syntax to allow conversation to progress in a decision tree. Patients were then provided with education about AF including lifestyle recommendations while also requiring the patient to respond to risk assessment questions. If a patient responded with concerning health status, they were referred to clinical care. No statistically significant difference was found between the AI and control groups, who received usual care, at 6 months. However, within the AI group, quality-of-life scores improved significantly, and 88.4% of participants reported the intervention as useful (Trivedi et al., 2025). Although primary clinical outcomes were not significantly different, the intervention demonstrated feasibility and high patient acceptability. Successful implementation of such a tool could potentially reduce travel demands and time constraints, increasing access to care for underserved populations.

Compared with high-income countries (HICs), low-and middle-income countries (LMICs) often face greater structural barriers to healthcare delivery, including workforce shortages, limited specialty care access, and constrained healthcare infrastructure (Kruk et al., 2018). Across LMIC settings, AI-based tools are increasingly being explored as cardiovascular decision-support systems with potential to expand access to specialty care in underserved communities. A recent study evaluating an AI model trained on echocardiographic data from Ugandan children and adolescents with latent rheumatic heart disease found that the model performed comparably to expert cardiologists in detecting mitral regurgitation (MR) and analyzing components of the MR jet characteristics to support rheumatic heart disease diagnosis (Brown et al., 2024). Similar AI-based diagnostic approaches have been investigated in countries such as China. In one study involving Chinese adults aged ≥40 years, investigators developed an ECG-based model to predict risk of developing cardiovascular diseases, including coronary heart disease, unstable angina, stroke and heart failure. The model demonstrated accurate prediction of 7-year CVD risk using ECG data independent of physical examination findings and laboratory measures (Zhou et al., 2025). In India, AI-enabled ECG has been explored as a strategy to improve diagnosis of acute cardiovascular events in underserved communities. A real-world hub-and-spoke implementation used AI-assisted ECG interpretation to facilitate earlier detection of acute coronary syndrome and significantly reduced turnaround times, reporting approximately 2.9 min for critical ECGs and suggesting improved identification of high-acuity ECG patterns (Chandra et al., 2026).

## AI and disease prevention

The application of AI-powered tools can help improve healthcare access and chronic disease management. A longitudinal study examined secondary CVD prevention medication use in 7,409 to 11,677 adults with CVD over a median of 12 years (2007–2019). It was found that medication used for secondary CVD prevention initially increased during initial follow up visit and overall utilization declined from 41.3% at baseline to 31.3% by the final visit (Joseph et al., 2025). Moreover, in a study of more than 16,000 U.S. adults, awareness and treatment of hypertension were generally high across all populations, but blood pressure control remained poor, particularly among minority groups. Black adults had higher rates of hypertension than White adults (45.3% vs. 31.4%, aOR, 2.24; 95% CI, 1.97–2.56; *p* < 0.001) and received treatment at similar levels, yet their blood pressure control remained worse (Aggarwal et al., 2021). A single-arm, nonrandomized study evaluated an AI-based hypertension management that integrated home blood pressure monitoring, wearables and lifestyle coaching through SMS messaging (Leitner et al., 2024). The system analyzed lifestyle and physiologic data to generate individualized behavioral recommendations and alert clinicals of critical readings. Among 141 participants monitored over 24 weeks, systolic and diastolic blood pressure decreased by 5.6 and 3.8 mmHg, respectively, at 12 weeks with further reduction at 24 weeks (SBP: −8.1 mmHg; DBP: −5.1 mmHg) compared to baseline. Participant engagement remained high with a mean weekly data collection of 92%, while only 5.9% of participants required manual outreach by a clinician. These findings suggest that AI-enabled remote coaching may support hypertension management with limited clinician intervention. However, interpretation is constrained by the study’s single-arm, nonrandomized design and lack of a comparator group. Additionally, observed blood pressure improvements may partly reflect a Hawthorne effect, whereby continuous monitoring and study participation influenced patient behavior independent of the AI intervention (Sedgwick and Greenwood, 2015). Although such approaches may improve access to care in resource-limited settings, careful attention to racial and ethnic disparities in treatment response, digital access, and algorithmic bias remains important to avoid reinforcing existing health disparities.

## AI and bias mitigation

Racial bias impacts treatment delivery to patients. A Study of Women’s Health Across the Nation (SWAN) investigated treatment of peri-menopausal Black, White, Asian, and Hispanic females with atherosclerotic CVD (ASCVD), T2DM, and HTN. Data showed that 90% of the women in the study with HTN were Black and Hispanic. Additionally, female individuals who met eligibility criteria for treatment of CVD with statins more likely to be Black or Hispanic. However, of the 808 females eligible for treatment of ASCVD with statin therapy, Black women had lower odds of reporting statin use (OR = 0.53, 95% CI: 0.36–0.78) (Jackson et al., 2020) This finding is troubling as it highlights that even though Black female individuals are eligible for treatments that will improve their disease outcomes, they do not receive them at the same rate.

Artificial intelligence may have the potential to reduce variability in clinical decision-making. However, its role in mitigating physician bias remains uncertain. In a randomized controlled vignette study, Goh et al., asked fifty physicians to evaluate video cases involving a White male patient with chest pain and Black female patient presenting with chest pain and to make clinical decisions regarding ECG interpretation, medications, and referral (Goh et al., 2025). After initial management decisions, the physicians were provided with clinical decision support generated by a GPT-4-based tool and allowed to revise their responses. The study found that physicians were willing to modify management plans following AI-assisted recommendations and assessment accuracy improved from 47 to 65% for the White male patient scenario and from 63 to 80% in the Black female patient scenario. However, these findings were derived from an experimental vignette design using simulated patient encounters and surrogate measures of clinical decision-making rather than real-world patient outcomes. Consequently, while AI-assisted decision support may help standardize aspects of clinical evaluation, its effectiveness in reducing bias or improving equity in routine clinical practice remains uncertain, particularly given the concerns regarding algorithmic.

## Ethical and implementation challenges

### Algorithmic bias

If AI systems intended to diagnose CVD are trained with datasets that contain bias, this may further increase the CVD risk among minority patients. AI is considered for use in clinical settings to minimize physician error. Paradoxically, inaccuracies within datasets will increase physician error.

A study investigated an AI model, which was trained utilizing a dataset in which over 80% of patients were White, for the analysis of cardiac MRI. The model was shown to generalize poorly across historically underrepresented populations, with performance dropping noticeably for Black, Asian, and other minority patients. This reduced accuracy led to substantially higher rates of incorrect heart failure classification in non-White patients, including more than a sixfold increase in misclassification among Chinese patients compared with White patients (Puyol-Antón et al., 2022).

These findings highlight the need for AI implementation to be approached with careful attention to equity, as doing so will strengthen and enhance the effectiveness of machine learning to predict disease outcomes and ultimately improve treatment delivery. Recent studies in machine learning and deep learning have sought to develop race-independent algorithms that minimize potential sources of bias. A convolutional neural network (CNN) deep learning algorithm was developed to identify patients with a left ventricular ejection fraction (LVEF) ≤ 35% from a 12-lead ECG. The CNN was trained on a predominantly homogeneous population (96.2% non-Hispanic White), raising concerns about generalizability given known racial and ethnic variation in ECG features. A separate CNN trained exclusively on non-Hispanic White participants was created for comparison, and a secondary analysis evaluated whether ECGs could be used to infer race. The CNN algorithm demonstrated consistent performance across all racial and ethnic groups (AUC ≥ 0.93), indicating that racial variation in ECG characteristics did not affect its ability to predict low LVEF (Noseworthy et al., 2020) This study highlights the possibility of making equitable AI algorithms that are able to accurately function across patient populations. Although this model functioned in a race-independent manner, minority populations such as Black/African American participants comprised only 1.2% of the study cohort, remained underrepresented. This underscores the need for greater data diversity to ensure that future AI models equitably reflect the populations they are intended to serve.

### Digital divide

Black patients with heart failure are twice as likely as White patients to have low health literacy, even after adjusting for demographics, comorbidities, social support, and insurance status (Chaudhry et al., 2011). Chinese and Hispanic patients likewise report lower health literacy, which may hinder effective cardiovascular self-management (Anderson et al., 2021). These challenges are often compounded by limited English proficiency and cultural barriers, which are associated with delayed care, lower use of preventive services, and poorer chronic disease management.

The integration of AI into cardiovascular care may further widen these gaps (Bailey et al., 2015). Many AI-enabled approaches rely on patient engagement with digital health technologies, including wearable devices, remote monitoring systems, and mobile health applications Patients with limited health literacy or digital literacy may have greater difficulty understanding, accessing or consistently using these tools, reducing their participation in AI-supported care models (Mackert et al., 2016). Because AI systems increasingly depend on continuous patient-generated data, individuals who are unable or unwilling to engage with them may be disproportionately excluded from the benefits of precision medicine (Babu et al., 2024).

Structural inequities in healthcare access present an additional concern. Underserved populations are less likely to receive care at academic or technologically advanced medical centers where AI-based diagnostic and therapeutic tools are most likely to be introduced (Kim et al., 2010). As AI becomes more integrated into healthcare delivery, disparities in digital access, health literacy, and institutional resources could reinforce existing health inequities rather than alleviate them. Achieving equitable implementation will therefore require targeted efforts to improve technological access, patient education, and digital literacy among disadvantaged populations.

### Privacy/security

Other ethical issues arise as sensitive medical record information may be susceptible to data breaches (Elendu et al., 2023). This is a consideration for technologists in the creation of these diagnostic tools. Employing methods to protect sensitive medical record information is imperative. Additionally, informed consent should be provided to patients whose data will be used for big data analysis as this maintains patient autonomy.

The most important consideration for AI utilization in health care is for it to be utilized in a beneficent manner. These AI models must not continue to perpetuate health disparities. In fact, it should be quality and highly intelligent to be useful to enhance care through more accurate diagnosis of cardiovascular patients. By expanding current cardiovascular research and creating more inclusive databases with intentionally diverse population information, this provides AI models with the data to ensure that AI technologies are quality, ethical, equitable, and accurate.

### Regulation

The FDA is the primary regulatory agency overseeing the implementation of AI in healthcare, evaluating AI-enabled technologies used in clinical care as medical devices. In this role, the FDA considers important criteria such as safety, effectiveness, and feasibility (Warraich et al., 2025). However, examination of the FDA’s role in regulating AI in healthcare remains important, as limitations in current regulatory processes may allow biases present in training and validation data to go undetected. A recent study reviewed 692 FDA-approved machine learning and artificial intelligence (AI/ML)-enabled medical devices Between 1995–2023 to assess sociodemographic representation among approved technologies. The FDA requires medical device manufacturers to provide a Summary of Safety and Effectiveness Data (SSED), which outlines the evidence supporting FDA approval (Muralidharan et al., 2024). The study found that 77.9% of SSEDs did not report demographic data and only 3.6% of SSEDs reported race and/or ethnicity. Furthermore, only 0.9% of approved devices reported information regarding socioeconomic status. Despite the low reporting of sociodemographic factors such as race, ethnicity, and socioeconomic status, cardiovascular devices accounted for approximately 70% of approved AI/Ml-enabled medical devices, representing the second highest among medical specialties (Muralidharan et al., 2024). These findings raise concerns regarding the potential for bias in AI-enabled cardiovascular care delivery and highlight the importance of demographic transparency during regulatory review. While these gaps may promote disparities in cardiovascular care provision currently, they also directly highlight areas where amendment in regulatory processes can be made to prevent further promotion of bias. Requiring manufacturers to report subgroup demographics within SSEDs and establishing standards for evaluating in model performance across race, ethnicity and socioeconomic groups may help reduce the risk of bias and promote equitable implementation of AI in cardiovascular care.

### Interpretation of AI diagnostic systems in practice

Although developing AI models have demonstrated diagnostic capabilities in cardiology, widespread implementation requires both interpretability and representative training data. Interpretability refers to clincians’ ability to understand how and why, and AI model arrives at a recommendation or clinical diagnosis. As AI models are integrated into clinical practice, interpretability enables healthcare providers to evaluate the diagnostic recommendations, identify potential biases, and determine whether recommendations are clinically meaningful, reliable, and equitable (Loh et al., 2022; Chaddad et al., 2023; Jung et al., 2023). This need for interpretability underscores the importance of training AI models on diverse and representative patient populations. Datasets that underrepresent certain racial, ethnic, or patients of a certain sex may produce biased recommendations, potentially perpetuating existing healthcare disparities (Ali et al., 2023). This bias can also impact clinician trust and confidence in AI-assisted decision-making, limiting integration and reducing the effectiveness of these tools in patient care.

## Conclusion

Artificial intelligence is rapidly transforming cardiology through advances in risk prediction, imaging interpretation, disease detection and remote monitoring. However, this technological progress exists alongside persistent inequities in cardiovascular care driven by structural barriers, underrepresentation in clinical datasets and unequal access to healthcare resources. This review demonstrates that although AI has substantial potential to reduce these disparities, it may propagate or amplify them when developed using biased datasets or implemented within unequal healthcare systems.

Importantly, many of these current AI applications remain proof-of-concept or early validation studies and substantial work is needed before widespread clinical adoption. Prospective studies are required to evaluate the validity, generalizability and real-world performance of these tools across diverse patient populations and healthcare environments (Kelly et al., 2019). Further research is also needed to address the logistical feasibility of scaling AI systems for clinical use, including workflow integration, clinical oversight, digital infrastructure, cost and long-term implementation outcomes.

The future impact of AI in cardiology will depend not only on model performance but also on how these technologies are designed, validated and deployed. Realizing this potential requires policies and initiatives that promote representative training datasets, transparent bias assessment, improved digital accessibility and intentional governance frameworks. Such measures may strengthen model generalizability while supporting inclusive and effective cardiovascular care. Without deliberate safeguards, AI risks reinforcing existing (Thamman et al., 2023). With thoughtful implementation, however it may become a valuable tool for advancing more accurate, accessible and equitable cardiovascular care.
